# Machine Learning-Powered Smart Sensing of Copper Ions in Water Based on a Carbon Dot-Incorporated Hydrogel Platform: An Easy Path from Bench to Onsite Detection

**DOI:** 10.3390/s26072142

**Published:** 2026-03-31

**Authors:** Ramanand Bisauriya, Richa Gupta, Ashwin S. Deshpande, Ansh Agarwal, Aryan Agarwal, Roberto Pizzoferrato

**Affiliations:** 1 Department of Electronics and Communication Engineering, Jaypee Institute of Information Technology, Noida 201309, India; ramanand.bisauriya@jiit.ac.in (R.B.); richa.gupta@jiit.ac.in (R.G.); r1ashwindeshpande@gmail.com (A.S.D.); anshgrwl2003@gmail.com (A.A.); 22102181@mail.jiit.ac.in (A.A.); 2 Department of Industrial Engineering, University of Rome Tor Vergata, 00133 Rome, Italy

**Keywords:** copper ion detection, colorimetric and fluorometric detection, water quality classification, risk-based thresholds, RGB color space, logistic regression, support vector machine

## Abstract

Water supplies contaminated by heavy metals pose a serious threat to human health, especially in areas without access to centralized testing facilities. While copper is a necessary heavy metal in trace levels, high concentrations can have detrimental effects on health, such as oxidative stress, cognitive impairment, and liver damage. Due to their expense, complexity, and reliance on laboratories, conventional detection techniques are accurate but unsuitable for real-time, dispersed deployment. Machine learning offers a potent solution to these constraints by facilitating the automatic, precise, and quick interpretation of complicated sensor data. It makes it possible to make decisions in real time without requiring a large laboratory infrastructure. In this work, a dual-mode optical sensor was developed using the colorimetry and fluorometry images of carbon dots embedded in hydrogels with the Cu^2+^ concentration of 0, 20, 50, 100, 200, and 500 μM. Data augmentation was used to expand the RGB picture dataset for each modality, and these data were interpolated to provide responses at 1 µM intervals (0–500 µM). We trained a comprehensive set of supervised machine learning models, including Logistic Regression, Support Vector Machines, Random Forest, and XGBoost, to categorize water samples into five risk-informed quality levels. The system achieved classification accuracies exceeding 96%. Furthermore, we built a simple user interface to make the system practically deployable in mobile phone. Together, these results demonstrate a scalable, interpretable, cost-effective, and quick solution for real-time water quality monitoring in resource-constrained environments. Since the proposed method focuses on classifying concentration ranges rather than precise quantification, a formal limit of detection (LOD) was not calculated; instead, the lowest concentration in the experimental dataset serves as the minimum detectable level.

## 1. Introduction

The expansion of global industrialization has intensified water pollution, particularly heavy metal contamination, which remains a persistent issue in global environmental protection. Heavy metals, such as copper (Cu), arsenic (As), lead (Pb), mercury (Hg), cadmium (Cd), chromium (Cr), and nickel (Ni), are bio-accumulative, non-degradable, and pose a significant threat to public health and environmental sustainability, ultimately endangering human health. Arsenic, a metalloid, is likewise a high-priority contaminant due to its toxicity and carcinogenicity. Mechanistically, arsenic acts as a protoplasmic poison, binding sulfhydryl groups and disrupting cellular respiration, enzyme activity, and cell division (Gordon & Quastel, 1948) [1]. Serious risks also come from other heavy metals, such as lead (Pb^2+^), a strong neurotoxin with significant effects on child development; mercury (Hg), which harms the nervous and renal systems; cadmium (Cd^2+^), which is linked to skeletal demineralization and renal dysfunction; hexavalent chromium (Cr(VI)), which has been shown to have carcinogenic potential; and nickel (Ni^2+^), which is linked to dermatological and systemic effects [2,3,4,5,6,7]. When creating monitoring plans and public health initiatives, it is essential to acknowledge these risks across various metals.

Copper (Cu^2+^) is one of these pollutants that has two sides: it is hazardous at high doses but necessary as a trace micronutrient. Long-term exposure to excess copper has been associated with oxidative stress, neurotoxicity, liver damage, and gastrointestinal issues [8,9]. Copper levels in water frequently rise above permissible limits in locations close to industrial zones, mining operations, and outdated infrastructure, putting impacted populations at grave risk [10]. The extent of the issue is demonstrated by real-world examples. Seasonal groundwater surveys in coal-mining corridors in Balochistan, Pakistan, have revealed exceedances of WHO guideline values for several parameters, including metals, in several locations [11]. Subsurface water samples taken close to mine workings in the Muslim Bagh mining area revealed copper concentrations as high as ~1.8 mg/L, with the highest concentrations occurring closest to active operations [12]. Butte, Montana, formerly known as “the richest hill on earth,” serves as an example of the long-term dangers associated with legacy mining in the United States. As the Berkeley Pit filled with acidic groundwater, dissolved heavy metals turned the region into a portion of the biggest superfund complex in the country [13]. When taken as a whole, these examples highlight how urgently vulnerable communities need decentralized, affordable, and field-deployable water quality monitoring devices. The U.S. Environmental Protection Agency (EPA) and the World Health Organization (WHO) set maximum permissible copper values of 1.3 mg/L (≈20.5 μM) and 2 mg/L (≈31.5 μM), respectively, to reduce risk [14].

The conventional detection techniques, such as Flame Atomic Absorption Spectrometry (FAAS) and Inductively Coupled Plasma–Atomic Emission Spectrometry (ICP-AES), etc., are highly selective and deliver high analytical accuracy, though they require large sample volume, laboratory infrastructure, and skilled personnel, and are also time-consuming, limiting responsiveness and real-time decision-making in the field [15]. To bridge this gap, optical sensing approaches, primarily colorimetric and fluorometric readouts, have gained traction for portability and cost-effectiveness. Colorimetric sensors yield visible color changes under white light, whereas fluorometric sensors track emission under UV excitation [16]. However, manual interpretation of these signals remains sensitive to illumination, background, and user bias. Recently, members of our group reported agarose-based dual-mode films incorporating o-phenylenediamine-derived carbon dots that respond to Cu^2+^ via both colorimetric and fluorescent channels, offering flexible readout options [17]. Yet, even with improved materials, human interpretation introduces variability that can hinder reliable field deployment.

With this article, we propose a machine learning (ML) framework that automates the classification of Cu^2+^ concentrations directly from images, unifying colorimetric and fluorometric modalities for rapid, scalable, and interpretable assessment. We compiled 5010 images per modality by interpolating six base concentrations (0, 20, 50, 100, 200, 500 μM) and applying geometric/photometric augmentations, yielding 501 discrete micromolar levels (0–500 μM). After benchmarking multiple color spaces for feature extraction, we selected RGB for its robustness and predictive performance in smartphone/digital colorimetry/fluorometric contexts [18,19]. We trained lightweight supervised classifiers, including Logistic Regression (LR), Support Vector Machine (SVM), Random Forest (RF), and XGBoost, and formulated the task as a five-class categorization aligned with practical decision bands for water quality based on copper concentration. An app with a graphical user interface (GUI) was created to facilitate non-expert usage. A layperson can use the smart app to upload an image and get results by just choosing the “analyze” option. The clear and straightforward outputs make the application highly convenient for practical on-site detection.

## 2. Background and Related Work

Recent research on copper-ion sensing has been clustered into two main approaches: electrochemical and optical systems. The electrochemical systems push sensitivity through signal amplification and learning-based decoding, while the optical systems (colorimetric/fluorometric) prioritize portability and low cost. A comparison summary of diverse detection techniques together with the ML framework and different parameters for copper ions and other targets has been listed in Table 1.

Fast-scan voltammetry (FSV) paired with deep neural models has demonstrated ultra-low limits of detection (LODs) by decoding complex voltammograms directly. FSV is impressive in sensitivity but typically constrained to bench-top, specialized instrumentation and controlled environments [20]. Optical approaches reduce hardware burden. For instance, a fluorometric pyoverdine-based probe that targeted the Cu^2+^ used the analytical calibration only with an ultra-low LOD, though it routinely reaches nanomolar LODs, but still requires a fluorimeter and careful calibration [21].

Smartphone-assisted colorimetry bridges lab and field by combining engineered chemobiosensors with image features and lightweight ML (e.g., SVM, RF, and LR), improving reproducibility and enabling portable analysis, yet sensitivity, ambient-light variability, and calibration drift can persist in real-world settings [22]. Across modalities, the literature shows a steady shift toward automated interpretation: from analytical curve-fitting and rule-based thresholds to ML classifiers and deep models that map signals (electrochemical or optical) directly to concentration estimates. Fully automated, risk-aligned multiclass outputs rather than binary decisions or raw regressions are only recently appearing, especially in dual-mode designs intended for variable field conditions [20,23].

**Table 1:** A comparative overview of ML-based copper ion detection methods.

Detection Approach	ML/Data Analysis	LOD, Linear Range (μM)	Automation & Usability	Shortcomings	Year & References
FSV with click-chemistry amplification (lab bench)	Deep CNN (FSVNet)	Single-atom Cu^2+^ detection (reported in the 10–16 μM regime; specialized ultra-low range)	Automated voltammogram analysis with very high sensitivity	Requires specialized electrochemical setup and controlled lab conditions; not field-portable	2024—[20]
Smartphone colorimetric chemo-biosensor	SVM, RF, LR on HSV image features	LOD: 0.09 ppm (≈1.416 μM) Cu^2+^ (low-μM regime); linear working range reported across low-ppm Cu^2+^ concentrations	Smartphone-based, portable platform; ML improves reproducibility and enables rapid on-site screening	Sensitive to ambient lighting and camera variability; lower sensitivity than lab-grade electrochemistry	2024—[22]
Fluorometric pyoverdine-based probe	Conventional analytical calibration (non-ML)	LOD: 50 nM (0.05 μM); linear fluorescence response in the low-μM Cu^2+^ region (≈0.2–10 μM)	Simple probe preparation with established Cu^2+^ selectivity	Requires a fluorimeter; manual, instrument-dependent readout; no ML component	* 2016—[21]
Co@Cu dual-metal electrochemical sensor (creatinine monitoring)	RF, Extra Trees, XGBoost	LOD: 130 μM and linear range defined for urinary creatinine (non-Cu analyte); high regression performance (R^2^ ≈ 0.98–0.99)	Low-cost printed electrodes; ML-assisted calibration and feature selection	Not a Cu^2+^ sensor; included only as an example of ML-guided electrochemical sensing	** 2025—[23]
Dual-mode RGB image sensor (colorimetric + fluorescent)	LR, SVM, RF, XGBoost	Five Cu^2+^ classes spanning 0–500 μM (studied concentration window)	Fully portable, Smart Phone-dual-mode imaging; direct image-to-class ML decision	Discrete band-wise classification rather than continuous concentration; affected by optical noise and imaging conditions	**Present work**

* Non-ML framework Cu^2+^ detection; ** non-Cu sensor, included for ML-workflow relevance.

## 3. Materials and Methods

This section outlines the complete experimental and computational workflow employed in our study. To ensure methodological clarity, we began by describing our previous sensing system [17,24], based on hydrogel films incorporating nitrogen and sulfur-doped carbon dots (NSCDs), and imaging conditions, followed by dataset preparation strategies, including interpolation and augmentation. All chemicals used throughout the experimental procedure were purchased from Merck Sigma Aldrich (Merk Life Science S.r.l., Milano, Italy) and all are of analytical reagent grade and used as received and the implementation work has been done on python open office named google colab (https://colab.research.google.com/). Subsequent subsections detail the preprocessing of images, exploration of color spaces, and the implementation of machine learning models used for copper ion concentration classification.

### 3.1. NSCDs Incorporated Hydrogel Films (Sensing System) Fabrication and Imaging

The sensing system utilized in this study was pre-acquired and published in our prior work [17,24], where o-phenylenediamine-derived nitrogen and sulfur doped carbon dots (NSCDs), which exhibit distinct colorimetric and fluorometric responses upon exposure to copper (Cu^2+^) ions, were incorporated in an agarose gel matrix. We studied how the two optical effects changed, thus achieving sensitivity to copper more easily by simple visual observation, even in real water samples. The overall behavior of the sensing system (SS) enabled two complementary optical (colorimetric and fluorescent) detection modes for copper ions in water.

The agarose–NSCD hydrogel films were prepared by dissolving 0.25 g of agarose in 10 mL of NSCD-sensing solution (2.5% (*w*/*v*)) at a pH adjusted to 9.5, followed by heating at 60 °C for 20 min to facilitate the oxidation reaction of o-phenylenediamine and to enhance the interaction between the sensing components and Cu^2+^ ions. This alkaline environment promotes the catalytic oxidation process and improves the optical response of the sensing system. The hydrogel film casting was made by pouring 2 mL of the above solution into 2 × 2 cm^2^ plastic cubes and leaving it at room temperature in atmospheric conditions to dry for 2 h. Finally, a square hydrogel film of average thickness of ~2 mm was easily removed.

**Figure 1:**
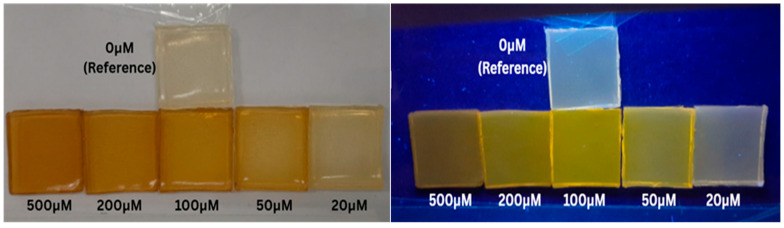
Visible change in the perceived color photographs (**left**: white daylight on a white background; **right**: a 365 nm UV light on a white background, illuminated) of six similar sensing films after a few minutes of immersion in water samples with different Cu^2+^ concentrations.

This strategy permitted easier testing procedures by simple immersion of the sensing film in water samples, even in a continuous mode, for a simple visual response. Figure 1 (left and right) displays two photographs (left: white daylight on a white background; right: a 365 nm UV light on a black background) of six similar sensing system films after 2 h soaking in water samples with different Cu^2+^ concentrations (10 μL of DI copper solution (CuCl_2_·2H_2_O) to reach the final ion concentration). It was reported that the concentrations as low as 10 μM can easily be detected both in daylight and especially under UV excitation due to a change in the perceived color within the first 10 min, with the optical response reaching full saturation after 2 h [17].

**Figure 2:**
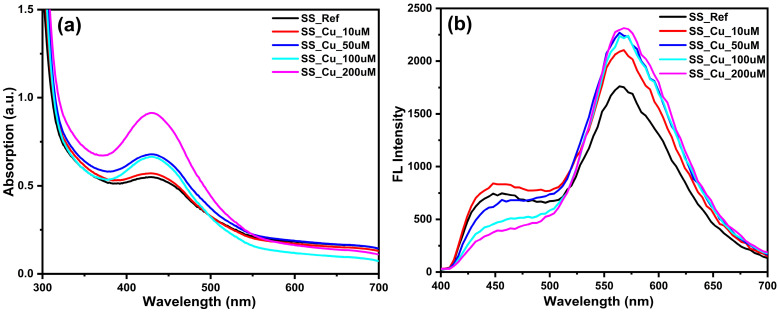
(**a**) Colorimetric and (**b**) fluorometric spectroscopy response of the above sensing system at different Cu^2+^ ion concentrations.

In order to understand the spectroscopic origin of this color change, absorption and fluorescence measurements were performed on the sensing films, as shown in Figure 2a and Figure 2b, respectively. The UV–Vis absorption spectrum of the sensor is reported in Figure 2a for different copper ion concentrations and shows the absorption band of oxidized o-phenylenediamine (OPDox) at 430 nm superimposed on the absorbance tail of agarose. Absorbance at 430 nm is enhanced after interaction with copper as a consequence of further oxidation of o-phenylenediamine by copper ions. Similar to absorption, the fluorescence spectrum of the sensing films (Figure 2b) resembles the superposition of the spectra of the two components, with the emission of OPDox at 564 nm not significantly affected by interaction with the solid matrix. Interaction with Cu^2+^ quenched the fluorescent UV/blue emission at 450 nm, and slightly enhanced the emission of OPDox at 564 nm [25,26]. The data, however, do not show a clear linear dependence on the ion concentration, and that is probably due to the relatively low reproducibility of absorbance and fluorescence measurements recorded over small areas of irregular soft samples, such as our agarose sensor. From this point of view, techniques based on image processing, such as the one we use in the present study, offer greater accuracy since the whole sensor surface is analyzed and becomes the foundation for our machine learning-based classification approach.

The image dataset served as the basis for the machine learning pipeline. From the original six base concentration images, additional interpolated samples were digitally generated to cover 501 unique micromolar levels between 0 and 500 µM. Each interpolated image was then subjected to nine augmentation transformations, including rotations, brightness adjustments, flips, and scaling, to simulate real-world variability. This resulted in a total of 5010 images per sensing modality, providing a large and balanced dataset for model training and evaluation.

### 3.2. Dataset Preparation

We created two parallel datasets corresponding to the dual sensing modes—colorimetric (white light illumination) and fluorometric (UV excitation)—in order to facilitate the classification of copper ion concentrations using supervised machine learning. According to the World Health Organization (WHO) and U.S. Environmental Protection Agency (EPA) global water quality guidelines, the sensor responses were initially recorded for six base copper ion concentrations: 0, 20, 50, 100, 200, and 500 µM, covering the range from safe to highly contaminated levels.

Fluorometric responses were recorded in the previous study [17] at UV excitation conditions appropriate for carbon dot fluorescence. We followed the same imaging circumstances during dataset development to ensure consistency.

A systematic two-stage data expansion technique was used because there were not enough base concentration images:

- **Interpolation**: Equation (1) illustrates how digital interpolation techniques were used to create intermediate images for each integer micromolar value between 0 and 500 µM. A smooth and fine-grained optical transition between known sensor responses was produced by this method, which produced 501 distinct concentration levels.

(1) $$I(c)=I(c_{i})+\frac{\left(c-c_{i}\right)}{\left(c_{i+1}-c_{i}\right)}\times (I(c_{i+1})-I(c_{i}))$$

where *I*(*c*) is the generated image at concentration *c*, and $c_{i}$, $c_{i+1}$ are the nearest known concentrations.

- **Augmentation**: To replicate real-world variability in imaging configurations, nine augmentation changes were applied to each interpolated image. These augmentations included the following:
  - Rotation by −5°;
  - Rotation by −10°;
  - Rotation by +5°;
  - Rotation by +10°;
  - Horizontal flipping;
  - Vertical flipping;
  - Brightness increase;
  - Brightness decrease;
  - Geometric scaling (cropping followed by resizing).

This resulted in 10 images per concentration level (1 original + 9 augmented), leading to a dataset of 5010 images per modality (colorimetric and fluorometric).

It should be noted that the augmented dataset was generated primarily through rotations and linear combinations of a limited number of experimental measurements. Although this strategy enables systematic evaluation of the tested methods, it may not fully reflect the variability present in real experimental datasets. Consequently, the reported performance metrics represent performance under controlled transformations, and further validation using larger datasets with greater experimental variability is required to assess the generalizability of the proposed approach.

To verify that the generated dataset preserves the optical response behavior of the sensor, we analyzed the mean RGB intensity trends across concentration levels. The RGB trends obtained from the interpolated datasets follow the same patterns observed for the experimentally measured concentrations in both colorimetric and fluorometric modes, as shown in Figures S1 and S2, respectively, and discussed in the Supplementary Materials. These validation plots indicate that the generated samples maintain the same concentration-dependent optical response trends.

**Figure 3:**
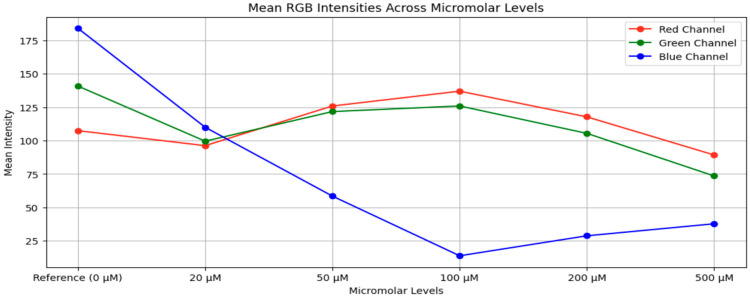
Mean RGB channel intensities for different concentrations of copper ions.

**Figure 4:**
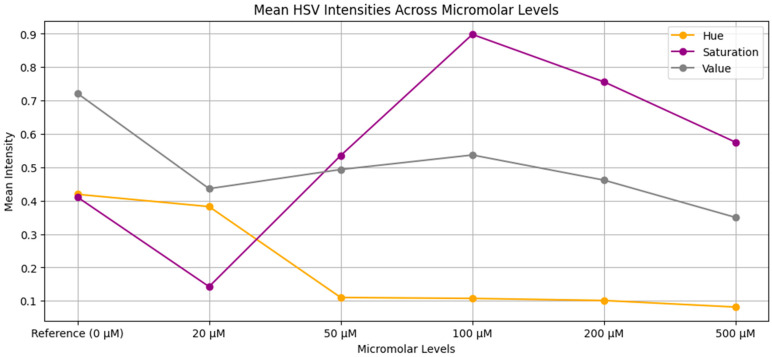
Mean HSV channel intensities at all levels of copper ion concentrations.

**Figure 5:**
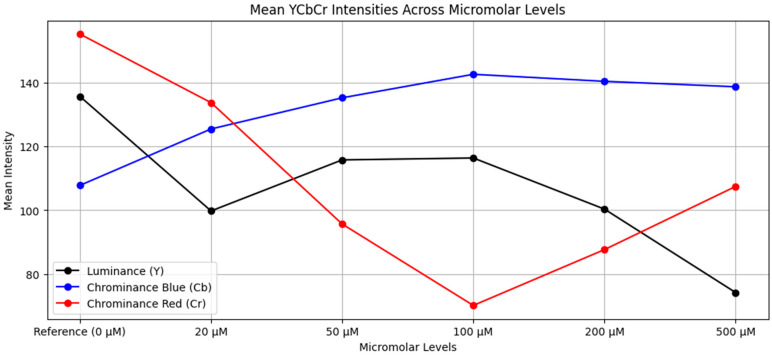
Mean YCbCr channel intensities at different concentrations of copper ions.

**Figure 6:**
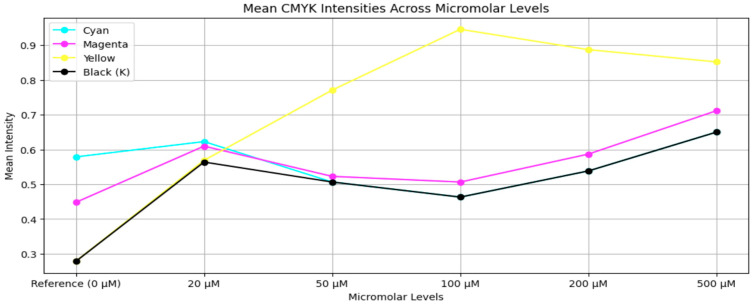
Mean CMYK channel intensities for all levels of copper ion concentration.

Despite these constraints, the controlled dataset construction enables a systematic evaluation of the fundamental predictive capability of the machine learning models before introducing additional experimental variability. We calculated the mean intensity values for specific channels (e.g., R, G, and B for RGB) across all samples in order to better understand the behavior of various color spaces within the copper ion concentration range, as indicated in Equation (2). Figure 3, Figure 4, Figure 5 and Figure 6 display the trends.

(2) $$\mu_{k}\left(c\right)=\frac{1}{N}\sum_{j=1}^{N}p_{j}^{\left(k\right)}\left(c\right)$$

where $p_{j}^{\left(k\right)}(c)$ is the pixel intensity in channel *k* (R, G, B, etc.) for images at concentration *c*, and *N* is the total number of pixels.

Color space comparisons were confined to the fluorometric pictures in order to streamline the evaluation procedure. It was deemed unnecessary to assess color spaces independently for both modalities because the colorimetric and fluorometric sensor pictures mostly captured changes in color intensity without significant structural variation. The fluorometric dataset was chosen at random for comparison. Following the finalization of the color model based on fluorometric images, the colorimetric dataset was also subjected to the same selection, which produced consistent and satisfying results.

RGB and HSV were determined to be the most promising options for more research after examining the mean channel intensity changes across several color spaces (Figure 3, Figure 4, Figure 5 and Figure 6) and assessing initial classification performances. While RGB demonstrated better accuracy and consistency, HSV provided theoretical benefits such as partial illumination invariance. Two extended datasets, one with RGB and the other with HSV representations, each with 5010 images per modality after interpolation and augmentation, were created in order to be sure thorough evaluation.

**Figure 7:**
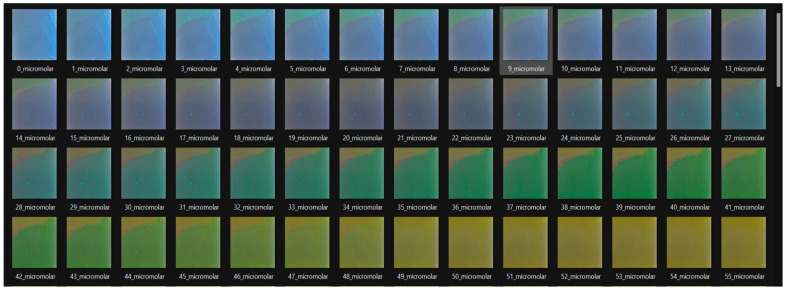
Artifacts encountered in images with HSV transformation. Note the presence of the green-tint distortions, which obscure important characteristics and impair model performance, particularly at lower copper concentrations.

The visual abnormalities caused by the HSV color space are shown in Figure 7; these are especially apparent in regions with high fluorescence intensities or at lower copper concentrations. Unwanted green-tint distortions are present in these images, which impair feature clarity and mislead the classification model. RGB images, on the other hand, more reliably maintained pertinent visual characteristics at all levels of concentration.

**Figure 8:**
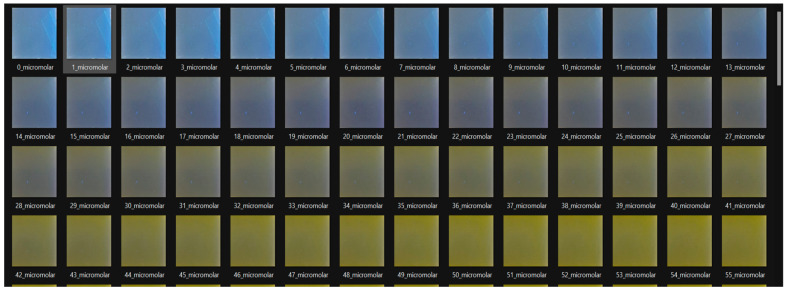
RGB-transformed dataset sample. Effective feature learning is aided by the image’s consistent visual quality and distinct differences throughout concentration levels.

When compared to RGB, YCbCr, and CMYK representations, the additional noise and visual inconsistencies caused a modest decrease in model performance, despite the theoretical advantages of color spaces like HSV. These findings led to the selection of RGB as the preferred color space for downstream modeling because of its greater categorization accuracy, stability, and ease of visual interpretation. A snapshot of the RGB dataset is shown in Figure 8, which enhances classification performance by demonstrating steady color constancy and a distinct visual evolution over concentration levels.

In this dataset, alternative color spaces like HSV introduced more noise than beneficial feature separation, despite their theoretical advantages. Because of its improved feature stability, higher classification accuracy, and better visual interpretability, RGB was chosen as the preferred color space for downstream modeling based on these comparative evaluations.

### 3.3. Machine Learning Models

We treat concentration estimation as multi-class classification across 501 classes (0–500 µM in 1 µM steps). Each sensing system image (colorimetric or fluorometric) was resized to 64 × 64 (RGB) and flattened; features were standardized by z-scoring computed on the training split to prevent leakage. Class labels preserve the ordinal structure but are learned in a standard multi-class setting. Cross-validation and final splits are detailed below (Protocol aligned with best practices in model selection and leakage avoidance) [27,28].

The following models were trained and evaluated:

- Logistic Regression [27];
- Support Vector Machine (SVM) [29];
- Decision Tree [30];
- Random Forest [31];
- XGBoost (Gradient-Boosted Trees) [32];
- K-Nearest Neighbors (KNN) [33];
- Gaussian Naïve Bayes [34];
- Multilayer Perceptron (MLP) [35].

To ensure a fair, well-controlled comparison across the model families described above, we used a consistent training and evaluation protocol. Model selection employed 6-fold stratified cross-validation to preserve class balance [28], and final results were reported on a stratified 80:10:10 train: validation:test split. All preprocessing (feature scaling and any target transformations) was fit strictly on the training partition to prevent leakage. Hyperparameters were tuned on CV folds–SVM (C, γ), Random Forest (number of trees, max depth, and max features), XGBoost (learning rate, number of trees, max depth, and subsampling/column sampling), KNN (k)(k), and MLP (width/depth, dropout, and weight decay), and the selected configurations were re-fit on train + validation, then evaluated once on the held-out test set. This design follows standard guidance on cross-validation and stratification [27,28].

#### 3.3.1. Evaluation Metrics

A variety of evaluation metrics, including accuracy, precision, recall (sensitivity), specificity, F1-score, and macro and micro averages, are used to assess the model’s performance [34,36].

To investigate adjacent-class confusions (e.g., 199 vs. 200 µM), which are important for judgments close to regulatory thresholds, we show confusion matrices; robustness against near-miss errors is highlighted by macro-F1 and per-class specificity [34,36].

#### 3.3.2. Practical Considerations and Justification

- **Calibration and risk bands**: Logistic Regression typically yields well-calibrated probabilities; tree ensembles can be post-calibrated if required (Platt/Isotonic) for risk-band decisions [27,32,34].
- **Interpretability**: Trees/RF/XGBoost provide feature importance and partial-dependence views that help iterate sensor design and understand failure modes [30,31,32].
- **Efficiency**: Naïve Bayes trains in milliseconds, KNN provides a fast separability probe, and SVM/boosting/MLP balance accuracy with runtime to enable edge-oriented deployment [29,32,35,37].
- **Data realities**: Class imbalance across 501 levels is mitigated by augmentation and stratification, and macro-averages prevent frequent levels from crushing rare ones [28,36].

## 4. Experimental Results

### 4.1. Model Performance Comparison

We evaluated several Supervised Models on both colorimetric and fluorometric image datasets to assess the classification accuracy of copper ion concentration levels. Five important measures were used to assess each model: accuracy, precision, recall, F1-score, and specificity. These are summarized in Table 2 and Table 3.

With an accuracy of 93.91%, precision of 94.70%, recall of 94.20%, F1-score of 94.20%, and specificity of 99.76%, the Support Vector Machine (SVM) outperformed all other models trained on colorimetric images. Following closely after were Random Forest (RF) and Logistic Regression (LR), both of which had a specificity of about 99.7% and an accuracy of over 92.5%. With an F1-score of 93.00%, the ensemble-based XGBoost classifier likewise demonstrated outstanding performance.

With accuracies of 88.76% and 86.95%, respectively, K-Nearest Neighbors (KNN) and Decision Tree demonstrated mid-tier performance. With accuracies just above 52%, the Multilayer Perceptron (MLP) and Naive Bayes models demonstrated limited efficacy, indicating weak applicability to this modality.

In every parameter, models trained on fluorometric pictures performed better than those trained on colorimetric images. With 96.77% accuracy, 97.15% precision, and an F1-score of 97.02%, SVM once again produced the best results. With comparable results (96.29% accuracy and 96.81% F1-score), Logistic Regression is a good contender because of its ease of use and interpretability.

With F1-scores of 95.42% and 96.01%, respectively, Random Forest and XGBoost classifiers trailed closely behind. These findings support ensemble approaches’ resilience when dealing with high-dimensional image data.

Notably, with accuracy levels below 53%, the Neural Network (MLP) and Naive Bayes models once more fared poorly. Even though they were trained on the same features, their decreased recall and specificity indicate that they had trouble learning from the image data.

### 4.2. Cross-Validation Insights

We used stratified six-fold cross-validation independently on the colorimetric and fluorometric datasets to make sure a reliable and objective assessment of all machine learning models. Given the fine resolution of the dataset over 501 different micromolar levels, this method preserved a balanced distribution of copper concentration classes across all folds.

Models were rotated through all possible permutations, trained on five folds, then verified on the remaining fold in each iteration. Equation (3) illustrates how the performance measures were then averaged over the six folds to reduce volatility and enhance reliability.

(3) $${Acc}_{CV}=\frac{1}{K}\sum_{k=1}^{K}{Acc}^{\left(k\right)},\;with\;K=6.$$

Top-performing models, especially Support Vector Machine (SVM) and Logistic Regression, showed low standard deviation in cross-validation findings. These models consistently achieved accuracy variations within ±0.5% across folds, demonstrating significant generalization capabilities. Because of their inherent stochastic components, ensemble techniques like Random Forest and XGBoost showed somewhat more unpredictability.

The improved signal-to-noise qualities and stability of fluorescence-based imaging are further supported by the fact that models trained on fluorometric data consistently beat those trained on colorimetric data in every fold.

**Figure 9:**
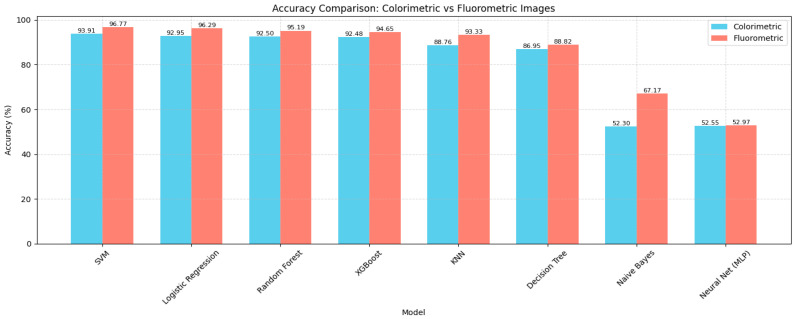
Accuracy comparison of all models trained on colorimetric and fluorometric datasets.

**Figure 10:**
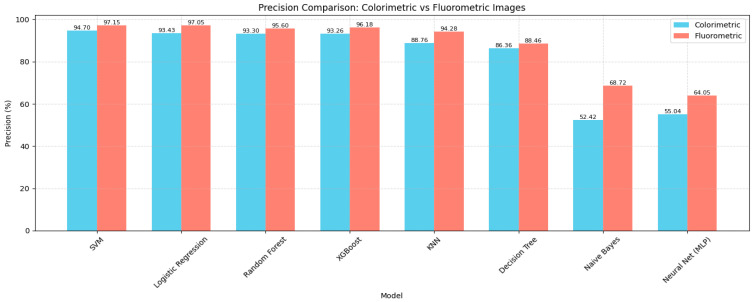
Precision comparison of machine learning algorithms for colorimetric and fluorometric image inputs.

**Figure 11:**
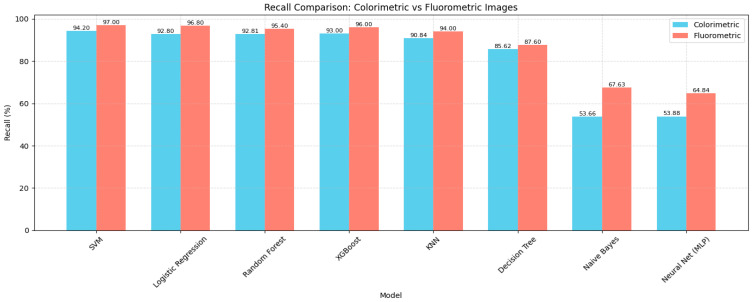
Recall model sensitivity comparison of colorimetric and fluorometric approaches.

**Figure 12:**
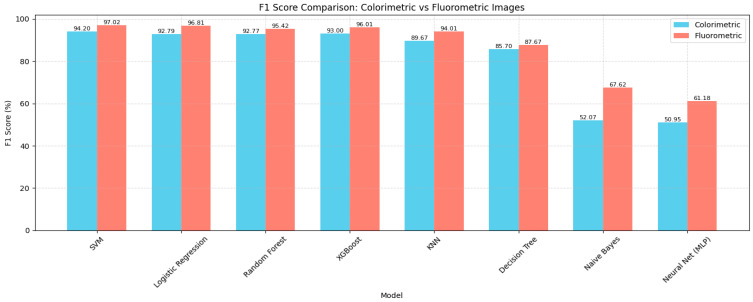
F1-Score comparison demonstrating each model’s overall balance between recall and precision across both modalities.

**Figure 13:**
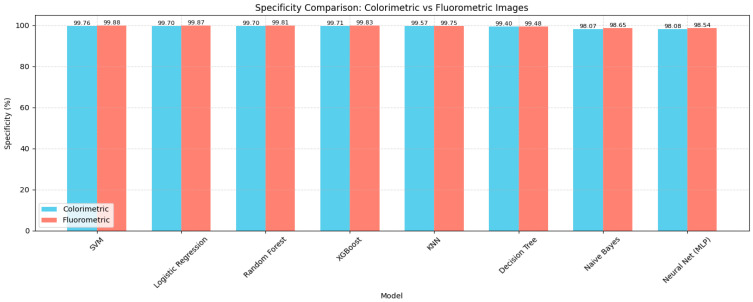
Comparison of the specificity of all classifiers trained on fluorometric and colorimetric datasets.

The comparative plots below (Figure 9, Figure 10, Figure 11, Figure 12 and Figure 13) show visual summaries of these cross-validation data, including accuracy, precision, recall, F1-score, and specificity, providing a clear and understandable picture of performance patterns across both sensing modalities.

## 5. Discussion

Experimental results clearly demonstrate the effectiveness of combining machine learning with dual-mode sensor imaging (colorimetry and fluorometry) for the detection of copper ions in water. Both Support Vector Machine (SVM) and Logistic Regression (LR) produced better results than more sophisticated models across all assessed criteria, as shown in Section 4.1. With 96.77% accuracy for fluorometric images and 93.91% accuracy for colorimetric images, SVM was the most accurate in both modes.

When compared to colorimetric imaging, fluorometric imaging consistently produced better findings. Higher signal contrast and fewer illumination artifacts in fluorescence-based measurements, which provide sharper pixel intensity gradients, are the main causes of this benefit. These improve the model’s capacity to pick up discriminative characteristics.

Even though ensemble techniques like Random Forest and XGBoost also did well, they only slightly outperformed SVM and LR. They might not be useful in real-time situations due to their greater computational demands. Multilayer Perceptron (MLP) and Naive Bayes, on the other hand, fared noticeably worse, especially in the colorimetric configuration, where their accuracies fell to about 52%, which is comparable to random guess levels. Over high-dimensional picture data, these models had trouble generalizing.

The most efficient color space was discovered to be RGB. It is better suited for training and interpretability than HSV or CMYK because it maintains spectral detail and structural consistency.

The five distinct water quality categories that were previously established in Table 1 (Section 1) served as the foundation for the classification system. By explicitly linking copper concentration ranges to certain risk classifications (such as Safe or Moderately Contaminated), this method makes interpretation easier. This categorization mapping makes things clearer and allows for quick action without the need for domain knowledge.

The dataset comprised 501 copper concentration classes (0–500 µM) with 10 augmentations each, yielding 5010 samples per modality to guarantee robust training. When combined with stratified six-fold cross-validation, this dense sampling enhanced generalization and decreased overfitting. We use SVM as the final model in both colorimetric and fluorometric datasets since SVM consistently performed better than LR.

### 5.1. Decision Thresholds and Operational Risk Banding

**Figure 14:**
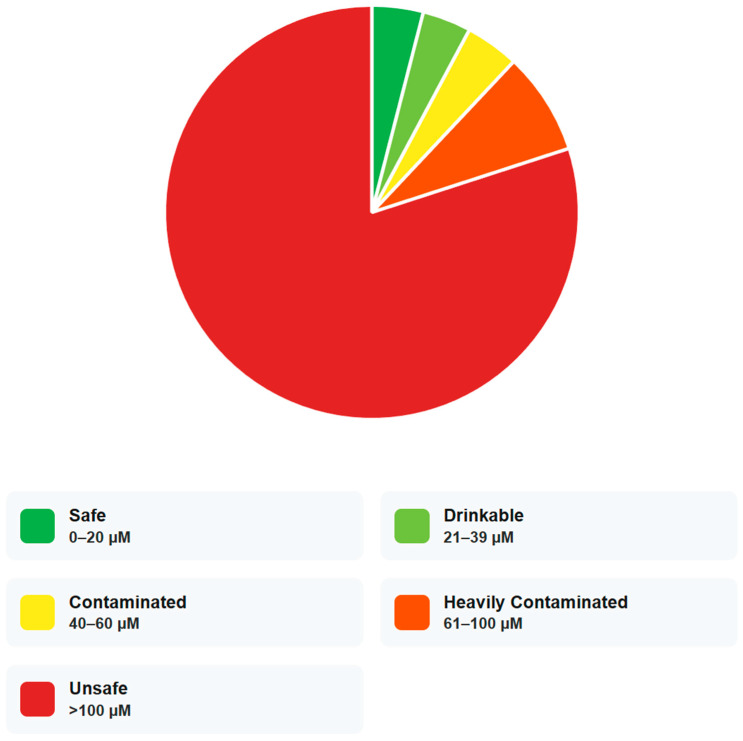
Copper contamination levels (µM).

We map anticipated concentrations to operational risk bands in accordance with practical tolerances found in our data and public-health recommendations (such as WHO/EPA criteria) [14] in order to convert model outputs into field decisions. Since the proposed framework focuses on classification of concentration ranges rather than precise quantitative estimation, the analytical detection limit was not explicitly determined. Instead, the minimum experimentally measured concentration included in the dataset represents the lowest detectable level considered for water quality classification. We use the bands listed in Table 4 and also presented in pictorial representation in Figure 14. In order to minimize the practical impact of adjacent-class confusions identified in our confusion-matrix research, we also advise marking predictions within ±1 µM of a boundary as “borderline” during deployment in order to initiate an automatic retest.

### 5.2. User Interface

#### 5.2.1. CuLens Mobile Edition—Interface Overview

**Scheme 1:**
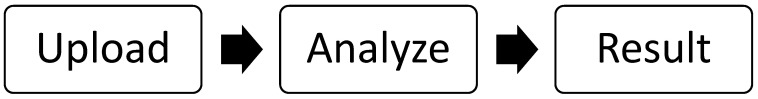
A workflow of CuLens.

The mobile edition of CuLens sustains the three important stages workflow as shown in Scheme 1.

These are updated for touch-based navigation and small screens. While preserving the desktop application’s scientific rigor, the interface guarantees readability, simplicity, and clarity.

**Figure 15:**
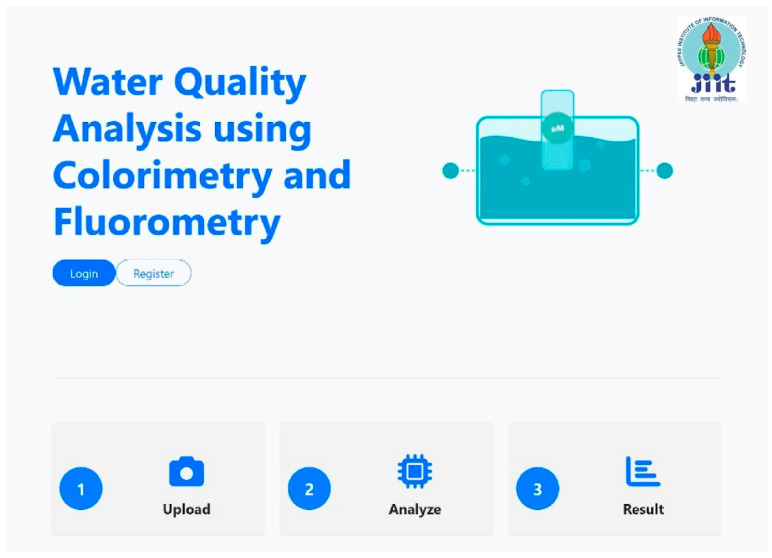
The project title, institutional logo, and stepwise navigation tiles (Upload, Analyze, and Result) with Login and Register choices are displayed on the CuLens Mobile Home Page.

When users first use the app, a clear landing page, as shown in Figure 15, that highlights the system’s goal—water quality analysis using colorimetry and fluorometry—is displayed. To help new users navigate the procedure, the three procedural steps are visually represented as tappable tiles. Quick access to authentication is made possible by prominent Login and Register buttons.

#### 5.2.2. Upload and Analysis Workflow

**Figure 16:**
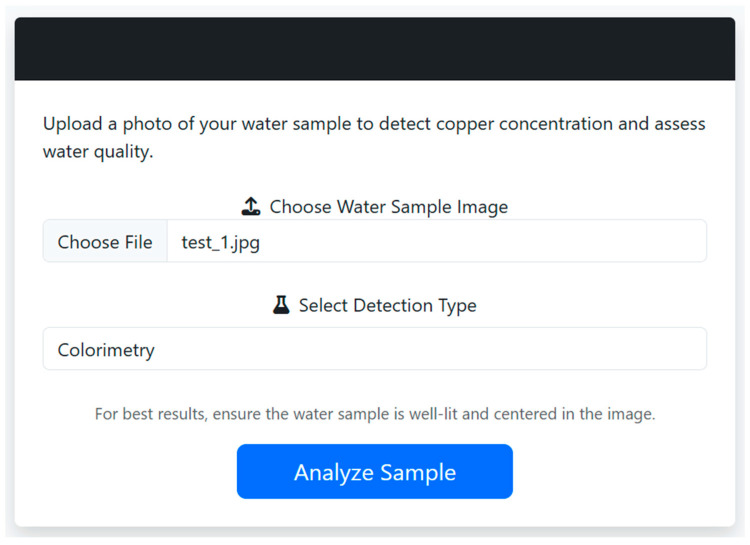
Mobile application upload and analysis interface showing file selection, detection-type dropdown, and analysis button.

Users can take or choose an image of their sensor test strip or water sample on the upload interface after logging in. The detecting method (colorimetry or fluorometry) can be chosen via a dropdown menu. The backend model inference process is started by clicking the Analyze Sample button. For accurate predictions, users should make sure the sample is well-lit, aligned, and easily visible, according to the instructional language beside the button shown in Figure 16.

Large input fields and single-tap actions are given priority in this mobile-optimized design, which enhances usability in field or lab settings. Every interactive element has real-time feedback, adaptive spacing, and is touch-friendly.

#### 5.2.3. Analysis Results and Interpretation

**Figure 17:**
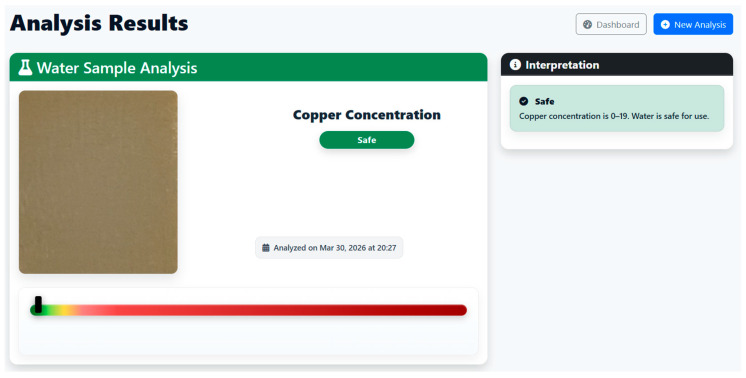
The “Safe” copper concentration classification is displayed on the Analysis Result screen along with a summary statement and matching color bar.

**Figure 18:**
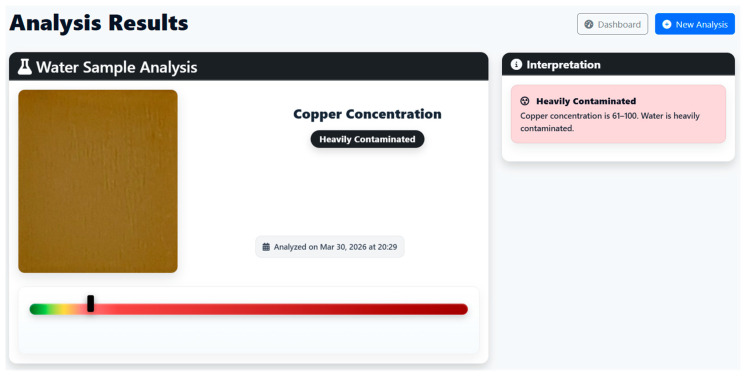
Analysis Results screen showing “Heavily Contaminated” classification and red-zone indicator on the contamination scale.

CuLens displays the analysis’s findings in a scrollable, vertically stacked card structure that works well on mobile screens. A classification badge, such as Safe, Moderate, or Heavily Contaminated, shows at the top after the examined sample image. Contamination severity is graphically communicated at a glance using a color-gradient scale (green → yellow → red) shown in Figure 17 and Figure 18.

Contextual text is included with every result, such as “Copper concentration: 0–19 μM” and “Water is safe for use”, as well as the analysis timestamp. For repeated testing, users can start a new analysis straight from the results page or return to the dashboard. High-contrast buttons, consistent typography, and a sparse use of color all contribute to the CuLens mobile interface’s uniformity. Every component is made to be accessible on all screen sizes and readable in a variety of lighting conditions. Users can easily collect, evaluate, and comprehend findings even on smaller devices because of the flexible layout.

## 6. Conclusions

The novelty of the present work lies in the integration of dual-mode optical sensing with modern machine learning-based image classification and a portable decision support interface, enabling more robust and practical field deployment. The system is based on the sensor platform described in a previous study, which uses o-phenylenediamine-derived carbon dots implanted in agarose matrices to record colorimetric and fluorometric responses to exposure to Cu^2+^ ions. In order to automate concentration classification, these optical changes were examined using a strong image-processing pipeline. Because the framework classifies ranges rather than precise concentrations, no explicit limit of detection LOD was calculated; instead, the lowest experimentally measured dataset concentration is taken as the minimum detectable level.

Among all the models trained on colorimetry and fluorometric images, the SVM with 96.77% accuracy, 97.15% precision, and an F1-score of 97.02% on fluorometry and an accuracy of 93.91%, precision of 94.70%, recall of 94.20%, F1-score of 94.20%, and specificity of 99.76% on colorimetric images outperformed all other models trained for both the color models. It performed better when evaluated using six-fold stratified cross-validation, especially on fluorometric data over colorimetric images because of the added features, whereas the Logistic Regression with 96.29% accuracy and 96.81% F1-score is also a good qualifier because of its ease of use and interpretability. In accordance with WHO criteria, the method divides water samples into five distinct risk groups instead of regressing continuous concentration results. This architecture enhances the solution’s interpretability and qualifies it for practical, field-level decision-making.

A graphical user interface (GUI) was created to facilitate non-expert usage, allowing end users to input sensing images and get immediate feedback on the state of water safety. The platform is appropriate for rural and resource-constrained situations because the user interface operates in offline conditions. The suggested system is a scalable option for decentralized water quality monitoring because of its excellent accuracy, affordable price, and field-ready interface. Future research will concentrate on adding more heavy metals to the detection range, carrying out practical validation tests, and incorporating the pipeline into mobile platforms for on-the-go diagnostics.

## Figures and Tables

**Table 2:** Comparative performance of the different models on the colorimetric dataset. SVM outperformed Logistic Regression and Random Forest in terms of overall metrics (93.91% accuracy, 94.20% F1-score). In colorimetry-based copper identification, MLP and Naive Bayes showed noticeably poorer accuracy, suggesting inadequate generalization.

Model	Accuracy (%)	Precision (%)	Recall (%)	F1-Score (%)	Specificity (%)
SVM	93.91	94.7	94.2	94.2	99.76
Logistic Regression	92.95	93.43	92.8	92.79	99.7
Random Forest	92.5	93.3	92.81	92.77	99.7
XGBoost	92.48	93.26	93.0	93.0	99.71
KNN	88.76	88.76	90.84	89.67	99.57
Decision Tree	86.95	86.36	85.62	85.7	99.4
Neural Network (MLP)	52.55	55.04	53.88	50.95	98.08
Naive Bayes	52.3	52.42	53.66	52.07	98.07

**Table 3:** Performance comparison across classifiers on the fluorometric dataset. Strong generalization was demonstrated by SVM and Logistic Regression, which performed better than others with F1-scores above 96%. Consistent with colorimetric results, ensemble models (XGBoost and RF) also demonstrated good robustness, although MLP and Naive Bayes trailed behind.

Model	Accuracy (%)	Precision (%)	Recall (%)	F1-Score (%)	Specificity (%)
SVM	96.77	97.15	97.00	97.02	99.88
Logistic Regression	96.29	97.05	96.80	96.81	99.87
Random Forest	95.19	95.60	95.40	95.42	99.81
XGBoost	94.65	96.18	96.00	96.01	99.83
KNN	93.33	94.28	94.00	94.01	99.75
Decision Tree	88.82	88.46	87.60	87.67	99.48
Naive Bayes	67.17	68.72	67.63	67.62	98.65
Neural Network (MLP)	52.97	64.05	64.84	61.18	98.54

**Table 4:** Operational risk bands for decision-making based on anticipated concentrations with borderline regions (±1 µM) triggering automatic retesting.

Copper Concentration (µM)	Category
0–20	Safe
21–39	Drinkable
40–60	Contaminated
61–100	Heavily Contaminated
>100	Unsafe

## Data Availability

The data and the images to carried out research will be available on suitable request.

## Supplementary Materials

The following supporting information can be downloaded at: https://www.mdpi.com/article/10.3390/s26072142/s1, Figure S1: Mean RGB intensity trends across copper concentrations (0–500 µM) for the colorimetric dataset. Error bars indicate standard deviation, and experimental points are shown as references.; Figure S2: Mean RGB intensity trends across copper concentrations (0–500 µM) for the fluorometric dataset. Error bars indicate standard deviation, and experimental points are shown as references.
