# Supplementary material for: Machine Learning-Powered Smart Sensing of Copper Ions in Water Based on a Carbon Dot-Incorporated Hydrogel Platform: An Easy Path from Bench to Onsite Detection
Source: Sensors (Basel). 2026 Mar 31;26(7):2142. doi: 10.3390/s26072142 (PMC13074651; doi:10.3390/s26072142)
Supplement: Supplementary file 1 [file sensors-26-02142-s001.zip › sensors-4173573-supplementary.pdf]

sensors

Article

Machine Learning-Powered Smart Sensing of Copper Ions in Water Based on a Carbon Dot-Incorporated Hydrogel Platform: An Easy Path from Bench to Onsite Detection

Ramanand Bisauriya[1], Richa Gupta[1], Ashwin S Deshpande[1], Ansh Agarwal[1], Aryan Agarwal[1], Roberto Pizzoferrato[2*]

[1] Department of Electronics and communication Engineering, Jaypee Institute of Information Technology, Noida, 201309, Uttar Pradesh, India.
[2] Department of Industrial Engineering, University of Rome Tor Vergata, 00133 Rome, Italy.
* Correspondence: pizzoferrato@uniroma2.it

Colorimetry:

We analyzed the colorimetric dataset by computing the mean and standard deviation of RGB pixel intensities for each concentration bucket (0–500 µM), with approximately 200 images per bucket. For every bucket, the R, G, and B channel values were averaged across all pixels and all images, and the corresponding standard deviation was calculated to capture variability within the dataset. These values were then plotted against concentration to obtain the RGB trend graph (Figure S1), where the curves represent mean intensities, and the error bars represent standard deviation. The experimentally captured images at six discrete concentrations (0, 20, 50, 100, 200, and 500 µM) were also plotted as reference points on the same graph. The extracted values show a consistent variation across concentration levels; for example, at 9.5 µM the RGB values were approximately R = 135.10 ± 8.88, G = 121.96 ± 9.40, B = 91.55 ± 11.27, while at 89.5 µM they were R = 131.12 ± 7.13, G = 94.17 ± 5.34, B = 25.19 ± 2.49. The resulting graph shows that the mean RGB trends follow a systematic pattern with concentration, and the experimental reference points lie close to these trends, while the error bars indicate the spread of values within each bucket.

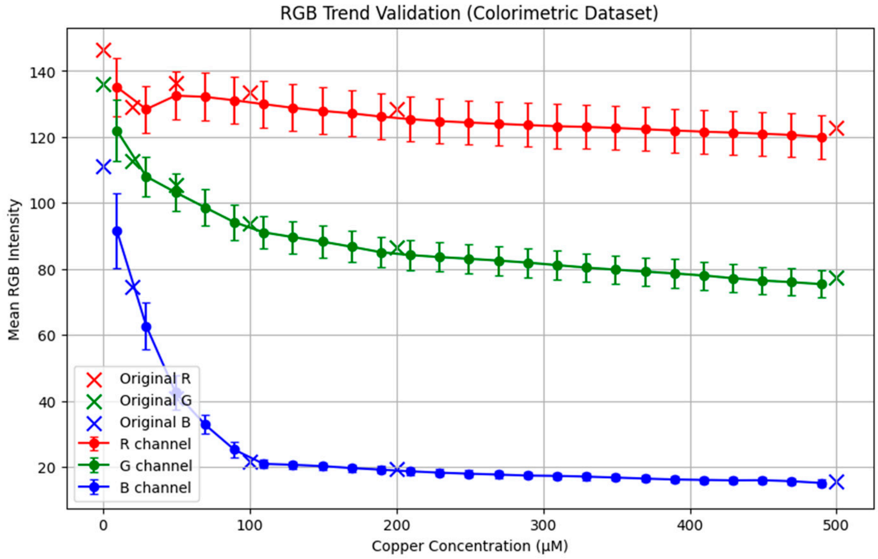

Figure S1. Mean RGB intensity trends across copper concentrations (0–500 µM) for the colorimetric dataset. Error bars indicate standard deviation, and experimental points are shown as references.

Fluorometry:

The RGB trend graph for the fluorometric dataset (Figure S2) represents the variation of mean pixel intensity values across the full copper concentration range (0–500 μM). For each concentration bucket, the mean and standard deviation of the R, G, and B channels were computed over all images (~200 per bucket). The plotted curves correspond to the mean values, while the error bars indicate the associated standard deviation, capturing variability within each concentration level. The experimentally captured images at six discrete concentrations (0, 20, 50, 100, 200, and 500 μM) are overlaid as reference points. The extracted values show a clear concentration-dependent variation; for instance, at 9.5 μM the RGB values were R = 98.70 ± 6.41, G = 117.38 ± 13.32, B = 144.79 ± 22.30, while at 89.5 μM they were R = 130.94 ± 7.36, G = 121.65 ± 6.70, B = 22.12 ± 5.22. The graph shows that the blue channel exhibits a sharp decrease with increasing concentration followed by stabilization, while the red and green channels initially increase and then gradually decline. The experimental reference points align closely with the mean trends, and the presence of non-zero variance across all levels reflects the distribution within each concentration bucket.

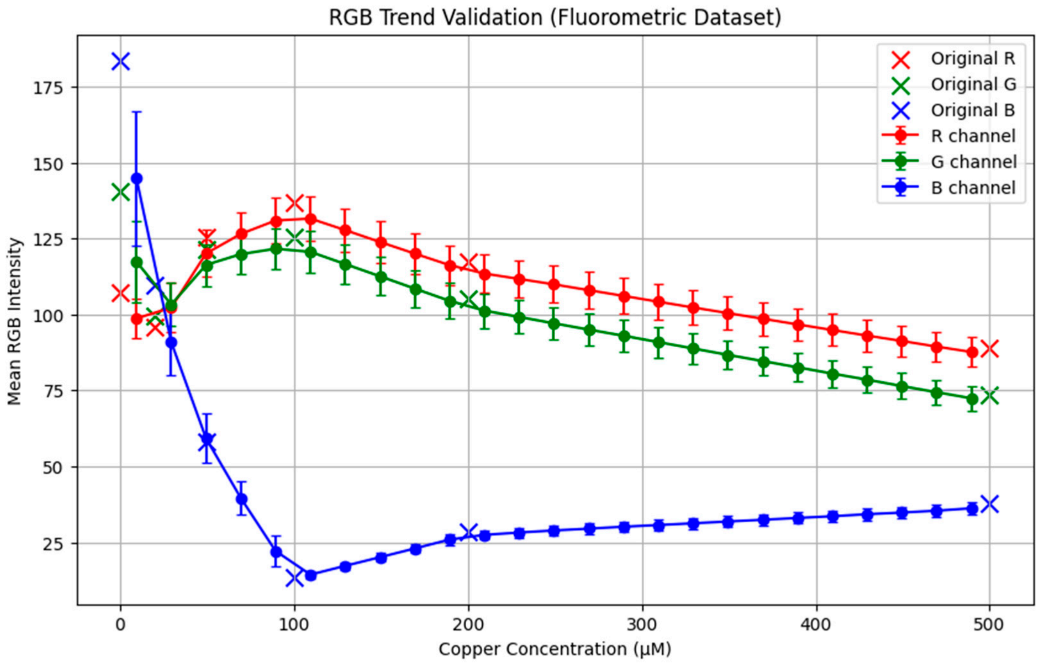

Figure S2. Mean RGB intensity trends across copper concentrations (0–500 μM) for the fluorometric dataset. Error bars indicate standard deviation, and experimental points are shown as references.
